# A Case in Which Calomel Excited the Symptoms of Poisoning by Corrosive Sublimate

**Published:** 1845-09

**Authors:** 


*A Case in which Calomel excited the symptoms of Poisoning by Corrosive Sublimate.*—Dr. Ashmead related the case of a patient under his care, to whom an ordinary dose of calomel was given as a purgative, and followed by a dose of magnesia—who, soon after taking the calomel, was seized with symptoms similar to those resulting from poisoning with corrosive sublimate. He was treated by the usual antidotes and remedies indicated in such cases, and recovered. There was no reason to suspect that the calomel given in this case was an impure article, or that there was any idiosyncrasy of constitution, as the patient had previously taken calomel without any unpleasant symptoms following.—*Transac. Coll. Phys. Phila.*

